# Development and Validation of the Breast Cancer Scale QLICP-BR V2.0 Based on Classical Test Theory and Generalizability Theory

**DOI:** 10.3389/fonc.2022.915103

**Published:** 2022-06-13

**Authors:** Fei Li, Jiali Zhou, Chonghua Wan, Zheng Yang, Qilian Liang, Weiqiang Li, Huanwei Chen

**Affiliations:** ^1^ School of Humanities and Management, Research Center for Quality of Life and Applied Psychology, Key Laboratory for Quality of Life and Psychological assessment and Intervention, Guangdong Medical University, Dongguan, China; ^2^ Medical Insurance Office, Capital Medical University Electric Teaching Hospital, Beijing, China; ^3^ School of Public Health, Guangdong Medical University, Dongguan, China; ^4^ Affiliated Hospital of Guangdong Medical University, The Three Wards of Medical Oncology, Zhanjiang, China; ^5^ Central Hospital of Guangdong Nongken, The Six Wards of Medical Oncology, Zhanjiang, China

**Keywords:** breast cancer, quality of life, classical test theory, generalizability theory, scale

## Abstract

**Objective:**

The aim of this study was to develop and validate the breast cancer scale among the system of quality-of-life instruments for cancer patients (QLICP-BR V2.0).

**Methods:**

Programmed decision procedures and theories on instrument development were applied to develop QLICP-BR V2.0. A total of 246 breast cancer inpatients were investigated using QLICP-BR V2.0 from hospital admission until discharge. The reliability, validity, and responsiveness of the QLICP-BR V2.0 scale were evaluated by using the classical test theory combined with the generalizability theory (GT), including correlation analysis, multi-trait scaling analysis, factor analyses, *t*-tests, and also multivariate generalizability theory analysis.

**Results:**

The test–retest reliability of the total scale is 0.79, the Cronbach coefficient is 0.85, and the intra-class correlations coefficient is 0.88. The item–domain correlation analysis showed that the correlation coefficient between items and their own domain is greater than that with other domains except of item GSO4. The exploratory factor analysis showed that three principal components are obtained in the specific module. The outcome of the factor analysis coincides substantially with our theoretical conception. The score difference of each domain of the scale and the total scale before and after treatment is statistically significant (*P* < 0.05), with the standardized response mean of the total scale being 0.61. According to GT, the generalization coefficient of the scores in the 5 domains is between 0.626 and 0.768, and the reliability index is between 0.557 and 0.695.

**Conclusion:**

QLICP-BR V2.0 exhibited reasonable degrees of validity, reliability, and responsiveness according to classical test and the generalizability theory. The number of items in the scale is appropriate.

## 1 Introduction

Breast cancer is one of the most common malignant tumors in women and an important obstacle to women’s health (1). In China, its morbidity and mortality are increasing year by year, accounting for 7 to 10% of various malignant tumors in the whole body. A recent report estimated that there were 4.3 million new cancer cases and 2.8 million cancer-related deaths in China in 2015, with breast cancer as the most common (estimated at 268,000 new cases) among women (2, 3).

With social and economic changes, the medical model has become a multi-dimensional concept containing multiple meanings of biology, psychology, and society. When we evaluate the treatment effect of breast cancer patients, we should no longer simply confine ourselves to survival time but should focus on whether patients receive adequate physical and psychological care during treatments (4). Many studies have shown that a scale can integrate the patient’s own feelings with clinical practice, and it is the core method for evaluating the health of patients, so many quality-of-life (QOL) assessment scales for breast cancer patients have been produced, including the following: European Organization for Cancer Research and Treatment Quality of Life Questionnaire-Breast Cancer Module (EORTC QLQ-BR23) (5), Functional Assessment of Cancer Therapy- Breast Cancer (FACT-B) (6), Hopwood Body Image Scale (7), which also focuses on the non-surgical treatment of breast cancer patients, and Body Image Questionnaire for Breast Cancer Patients (BIBCQ) (7), which does not solve the esthetic problems after breast reconstruction. Among them, EORTC QLQ-BR23 and FACT-B are the most widely used, both for surgical treatment and/or non-surgical treatment of breast cancer patients, and are disease-specific rather than surgery-specific measurement tools. However, most scales are mainly suitable for European and American environments for QOL cultural dependence. Although the Chinese versions of QLQ-BR53 (8) (QLQ-C30 and QLQ-BR23) and FACT-B (9) can be used for Chinese patients, they are lacking Chinese cultural backgrounds to some extent considering their original use in English-speaking patients—for example, the QOL scales developed abroad are constructed mainly under the Western cultural system, which are more concerned about the two aspects of religious belief and sexual life (10).

In China, Peng *et al.* (11) compiled a questionnaire for evaluating the quality of life of breast cancer patients, including 64 items in 4 dimensions of physical, psychological, symptom, and social function. However, the development of this scale has not been updated for a long time and not based on modern test theory. Zhang *et al.* (12) formulated the (Patient Reported Outcome scale of Chinese medicine after breast cancer surgery, but this scale has not been tested for test–retest reliability and may not achieve long-term efficacy evaluation. Moreover, the responsiveness of the scale (the differences before and after treatments) needs to be investigated further. It is necessary to develop Chinese-specific QOL instruments systematically. In response to this need, our QOL team started the research focusing on the development of the quality-of-life scales for cancer patients since 1997. The Chinese QOL instrument system called Quality-of-Life Instruments for Cancer Patients (QLICP) was developed by module approach. This system includes a general module (QLICP-GM) which can be used with all types of cancer and specific modules for different cancers, with each module being used for only the relevant cancer. The first version of the system has been completed in 2013, with 13 scales being developed, including the QLICP-GM and the 12 cancer-specific QOL instruments such as those for lung cancer, head and neck cancer, colorectal cancer, *etc.* (13–16). The first version of the breast cancer scale QLICP-BR V1.0 is an important one of this system and has been put into use after it has been developed (17, 18).

However, QLICP-BR V1.0 also exposed some problems during long-term use. Firstly, the specific module mainly describes the specific adverse reactions of breast cancer, which need to be distinguished from other diseases. There may be insufficient item expression, and the structure of the scale may need to be adjusted. Secondly, with the improvement of medical technology, there may be new changes in the specific response of the disease in the specific module, and items need to be added or deleted. Thirdly, theoretical support needs to be updated. The theoretical basis for the development and validation of the first version of the system is mainly classical test theory (CTT), and it still has some shortcomings. The modern test theory should be fully combined with the scale development.

Therefore, we have started the second version of the system QLICP V2.0 since 2010 based on V1.0 and in accordance with classical test theory and modern test theories such as generalizability theory (GT). QLICP V2.0 includes the general scale (module) QLICP-GM V2.0 and 22 cancer-specific scales such as those for brain cancer, bladder cancer, prostate cancer, cervical cancer, leukemia and lymphoma, *etc.* Up to now, most scales of the QLICP V2.0 have been developed and put into use (19). This paper is aimed to report the developmental process and validation of QLICP-BR V2.0, which will assist in management and decision making (19) and has also wide practical applications because patients with breast cancer account for a large proportion of cancer cases in China and also in the world.

## 2 Materials and Methods

### 2.1 Patients

This study is based on inpatients with breast cancer clinical diagnosis and diagnosed by pathological examination in the Affiliated Hospital of Guangdong Medical University and Central Hospital of Guangdong Nongken. The inclusion and exclusion criteria are as follows:

–Inclusion criteria: (1) patients with a clear diagnosis, that is, those diagnosed as breast cancer by pathological examination; (2) good reading and presentation skills and able to fill out questionnaires by themselves; and (3) volunteered to participate in the survey—no mental illness or disturbance of consciousness.

–Exclusion criteria: (1) cognitive and consciousness dysfunction; (2) those who refuse to participate in the research or those with a low degree of education; (3) combined with other primary cancers, other serious diseases, mental illnesses, *etc.*; and (4) multiple metastases of malignant tumors.

### 2.2 Development of QLICP-BR V2.0

**Figure 1:**
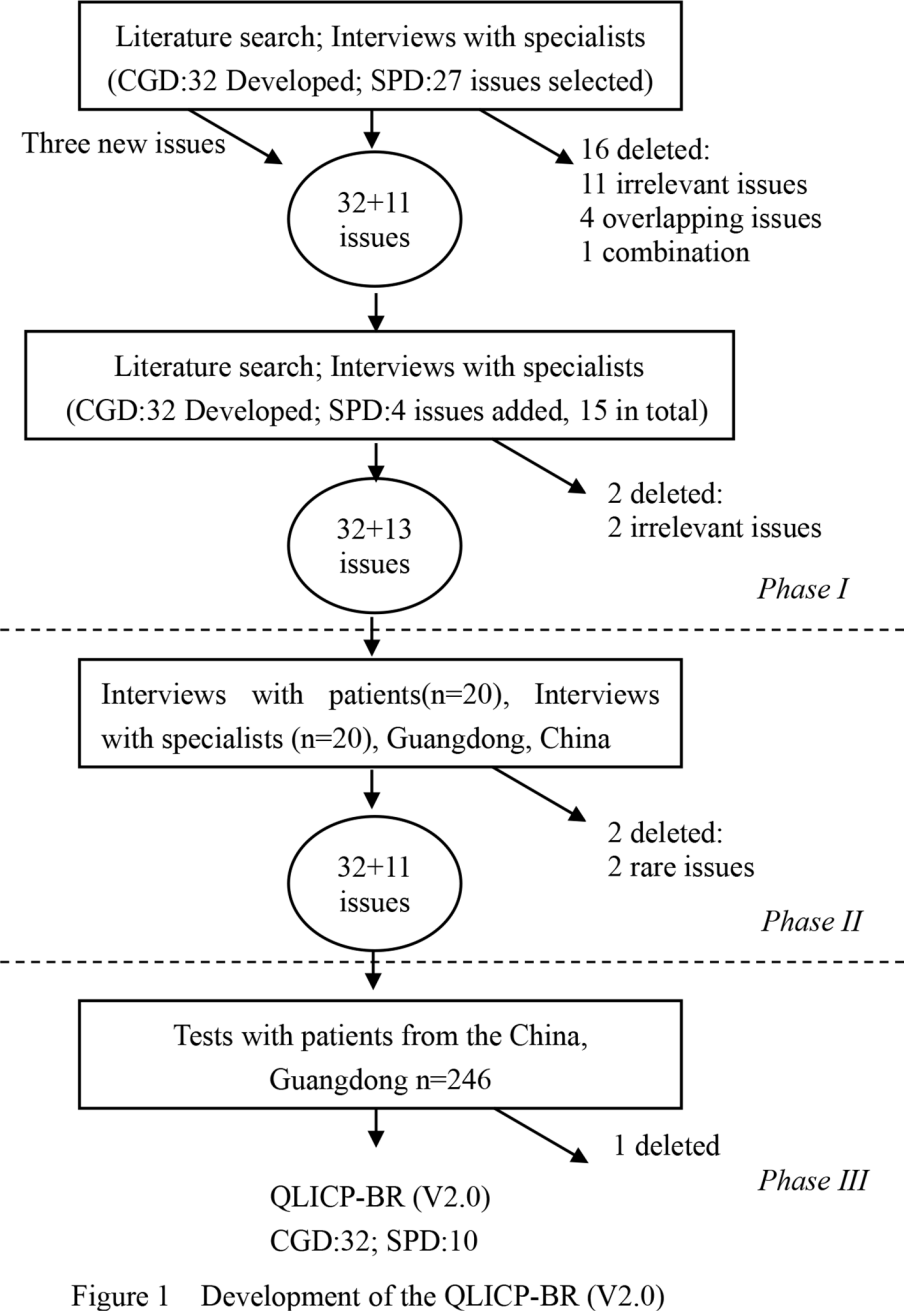
Development of the QLICP-BR (V2.0).

The scale adopts the modular approach by combining the general module with the specific module for breast cancer. The methodology is similar with that of the first version (17, 18), and the main steps to form the final QLICP-BR V2.0 are presented in **Figure 1**.

The general module QLICP-GM V2.0 has been successfully developed and was confirmed to have good reliability, validity, and responsiveness (20) in 2015 using classical test theory, item response theory, and generalization theory. It includes 4 domains and 32 items: physical, psychological, social, and the common symptoms/side effects.

The specific module of QLICP-BR V2.0 is developed in strict accordance with the following steps:

(1) Establish a research team, which includes experts and scholars in the domains of quality of life, statistics, public health, psychology, and breast cancer.
(2) The conceptual framework of the specific module of breast cancer patients is presented according to the definition of QOL, which can be classified into 3 facets: clinical symptoms, treatment side effects, and specific psychological effects.
(3) The formation of the item pool of the scale is mainly based on the decomposition of the multidimensional QOL concept of breast cancer, the search of literature, the reference of domestic and foreign mature scales, and the clinical experience of breast cancer. As a result, 24 items in the item pool are proposed under the abovementioned three facets.
(4) Selection and determination of items: Through discussions by the experts of the research group, the items that have no significant impact on the quality of life of breast cancer patients were deleted to form a preliminary scale. During this process, this study conducted two rounds of discussions, and finally 13 items were kept to determine the specific module, as shown in **Figure 1**, phrase I.
(5) Pre-survey and item selection. Using the preliminary scale, a questionnaire survey of patients was conducted, and item selection was conducted by statistical analysis and also experts’ clinical experience. After the discussion by experts at this stage, 11 items were selected to form a test version of the scale, as shown in **Figure 1**, phrase II.
(6) Item selection again based on a survey using a test version. A questionnaire survey using a test version of the scale was conducted, and an item was screened again by statistical analysis and also experts’ discussions. As a result, 10 items were selected to determine the official version of the scale, as shown in **Figure 1**, phrase III.
(7) Evaluation of the formal version of the scale: This study uses classical test theory and generalization theory to evaluate the reliability and validity of the scale.

### 2.3 Evaluation of QLICP-BR V2.0

The specific module of the breast cancer was combined with the general module to form the complete QLICP-BR V2.0. A large-scale questionnaire survey was conducted among eligible breast cancer patients to validate the QLICP-BR V2.0.

#### 2.3.1 Survey Methods

The investigator (doctors, nurses, and medical postgraduate students) briefly explained the content and purpose of the investigation. After obtaining the consent of the patient and the signed informed consent form, the investigator sent the QLICP-BR V2.0 to the patients to fill out by themselves. The first questionnaire survey was conducted on the first day after admission, the same questionnaire was used for the retest survey on the second or third day after admission to evaluate the test–retest reliability, and the third survey was conducted before discharge in order to evaluate responsiveness.

#### 2.3.2 Scoring Methods

Firstly, the raw scores (RS) of items, domains, and overall scale were calculated according to the unified scoring rules on the scale. Each item of QLICP-BR V2.0 is rated in a five-level scoring system, namely, not at all, a little bit, somewhat, quite a bit, and very much. Therefore, the positively stated items directly obtain scores from 1 to 5 points, and the negatively stated items are reversed. Each domain score is obtained by adding its own item score together, and the overall scale score is the sum of five domain scores.

Secondly, the corresponding standard score (SS) for all domains and the overall were linearly converted to a 0–100 scale using the formula: SS = (RS - Min) × 100/R, where SS, RS, Min, and R represent the standardized score, raw score, minimum score, and range of scores, respectively.

After scoring, classical test theory and modern test theory were used to evaluate the validity, reliability, and responsiveness of QLICP-BR V2.0.

#### 2.3.3 Scale Assessment Based on Classical Test Theory

##### 2.3.3.1 Reliability Assessment

We evaluate the reliability of the scale by calculating the test–retest reliability, Cronbach’s *α* coefficient, and intra-class correlation coefficient (ICC) and its corresponding 95% confidence interval (21).

##### 2.3.3.2 Validity Evaluation

The validity of content was evaluated by means of expert evaluation. Construct validity was evaluated by calculating the Pearson correlation coefficient, *r*, among items and domains as well as factor analysis. Exploratory factor analysis was used to examine whether the scale structure is consistent with the theoretical conception (22). In this study, the Chinese version of FACT-B was selected as the criterion for assessing the criterion-related validity, and the correlation coefficient between the domain scores of QLICP-BR V2.0 and FACT-B (V4.0) was calculated.

##### 2.3.3.3 Responsiveness Evaluation

The average scores between the first and third assessments (before and after treatments) were compared by paired *t*-test, with the standardized response mean (SRM) being calculated, which is the ratio of the difference before and after treatment to its standard deviation.

#### 2.3.4 Scale Assessment Based on Modern Test Theory

GT is a modern measurement theory that introduces irrelevant variables or factors that interfere with test scores into the measurement model and analyzes the impact of these factors and the interaction between factors and factors on the measurement scores through statistical techniques. It is applied in quantitative research to analyze the influence of patients, items, and interactions between patients and items on the total score of the scale. GT provides a comprehensive and unifying framework that goes beyond the CTT model of a single error term by allowing for the simultaneous analysis of the main and interaction effect source of error variance (23, 24). GT subsumes other forms of reliability approaches (*e*.*g*., internal consistency reliability, inter-rater reliability, and intra-class correlation) and provides a comprehensive and unifying framework for assessing the measurement reliability, especially for complex measurement situations. The application of GT includes the univariate generalizability theory method and the multivariate generalizability theory (MGT) method. The MGT was initially proposed by Cronbach based on the multivariate analysis of variance, and it is appropriate for multidimensional and complicated measurement situations (24).

The GT-based scale assessment includes G-study and D-study: (1) G study, also known as generalizability study, has the main task to find out various potential sources of measurement errors in the research design as much as possible in the universe of admissible observations and to estimate the variance components of these error sources; and (2) D-study, also known as decision research, has the main task which is based on G-study by adjusting various relationships in the measurement process to explore how to control and adjust measurement errors. Its indicators are generalization coefficient and reliability index (25, 26).

### 2.4 Data Analysis Software

In this study, SPSS25.0 was used to calculate the reliability, validity, and responsiveness, and mGENOVA was used for generalizability theory analysis.

## 3 Results

### 3.1 Socio-demographic and Clinical Characteristics of Breast Cancer Patients

A total of 246 breast cancer patients were investigated in this study, all of which were women. Moreover, these patients range in age from 17 to 77, with a mean age of 50.07 ± 10.25, and 96.3% (237 cases) were of Han ethnicity. The household economy is mostly medium, accounting for 67.9% of the total population. In terms of occupation, workers accounted for 8.1% (20 cases) and 45.5% (112) were farmers. Furthermore, 97.2% were married. A total of 148 cases (60.2%) finished middle school or high school, while 65 (26.4%) completed primary school and 33 (13.4%) had a college/university degree. In addition, 226 cases (91.9%) used medical insurance, while 20 cases (8.1%) used self-paid/private insurance. On the basis of clinical stage, 53 cases (21.5%) were in stage I, 86 cases (35%) were in stage II, 54 (22.0%) were in stage III, and 27 (11.0%) were in stage IV.

### 3.2 Evaluation Results Based on Classical Test Theory

#### 3.2.1 Reliability

In this study, the test questionnaires on the day of admission of the breast cancer patients and the second or third day of admission were tested for test–retest reliability. The results show that the test–retest reliability of each domain is greater than 0.8 (the ideal value is greater than 0.7 (27)), and the test–retest reliability of each facet is between 0.7 and 0.8. **Table 1** shows a summary of the test–retest reliability.

**Table 1:** Reliability of the quality-of-life instrument QLICP-BR (V2.0) (*n* = 246).

Domains/facets	Internal consistency Coefficient *α*	Test–retest reliability correlation *r*	ICC (95%CI)
Physical domain	**0.63**	**0.86**	**0.92** (**0.89**–**0.95**)
Basic physiologic functions	0.64	0.87	0.93 (0.90–0.95)
Mobility and mobility	0.56	0.80	0.89 (0.84–0.92)
Psychological domain	**0.77**	**0.81**	**0.89** (**0.85**–**0.93**)
Cognition	0.59	0.85	0.92 (0.89–0.94)
Emotion	0.64	0.79	0.88 (0.83–0.92)
Will and personality	0.57	0.80	0.89 (0.84–0.92)
Social domain	**0.63**	**0.82**	**0.90** (**0.86**–**0.93**)
Interpersonal communication	0.03	0.76	0.86 (0.80–0.90)
Social support and security	0.08	0.77	0.87 (0.81–0.91)
Social role	0.44	0.84	0.91 (0.88–0.94)
Common symptoms and side	**0.66**	**0.85**	**0.92** (**0.88**–**0.94**)
effects			
Common symptoms	0.76	0.87	0.93 (0.90–0.95)
Common side effects	0.46	0.74	0.85 (0.79–0.90)
Sub-total	**0.91**	**0.80**	**0.89** (**0.84**–**0.92**)
Specific domain	**0.74**	**0.83**	**0.91** (**0.87**–**0.94**)
Clinical symptoms	0.81	0.86	0.93 (0.89–0.95)
Therapeutic side effects	0.63	0.77	0.87 (0.82–0.91)
Specific psychological effect	0.77	0.87	0.93 (0.90–0.95)
Total	**0.85**	**0.79**	**0.88** (**0.83**–**0.92**)

ICC, intra-class correlation; CI, confidence interval. Bold values represent results for domains of the scale. Other values represent results for facets of domains.

The internal consistency reliability of the scale was assessed through Cronbach’s *α* coefficient (Cronbach’s *α*) and ICC. The result shows that Cronbach’s *α* coefficient of the total scale is 0.85 (the ideal value is greater than 0.7 (17). At the same time, the Cronbach’s *α* coefficients of various domains of the general module and the specific module are all around 0.7. In addition, the ICC values for these five domains were higher than 0.85. **Table 1** shows a record of the details of Cronbach’s *α* and ICC.

#### 3.2.2 Validity

QLICP-BR V2.0 is based on a large amount of literature review and many discussions by experts in the subject group. It involves physical function, psychological function, social function, common symptoms and side effects to cancer patients, and the specific symptoms and special psychological changes of breast cancer patients. Through rigorous procedures and methods, the items are also screened and analyzed. These insure good content validity.

The construct validity was evaluated by item–domain Pearson’s correlation coefficient *r*. As shown in **Table 2**, with the exception of item GSO4, there is a strong correlation between the items and their domain (mostly above 0.40). However, the relationship between the item and the other domains is weak.

**Table 2:** Correlation coefficient *r* among items and domains of QLICP-BR (V2.0) (*n* = 246).

Code	Items brief description	Physical	Psychological	Social	General module	Specific module
GPH1	Appetite	**0.69** ^a^	0.33^a^	0.20^a^	0.39^a^	0.17^a^
GPH2	Sleep	**0.60** ^a^	0.17^a^	0.06	0.24^a^	0.04
GPH3	Sexual function	**0.25** ^a^	0.16^b^	0.16^b^	0.02	0.01
GPH4	Excrement	**0.67** ^a^	0.23^a^	0.21^a^	0.25^a^	0.10
GPH5	Ability of daily living	**0.62** ^a^	0.47^a^	0.46^a^	0.39^a^	0.21^a^
GPH6	Positive and optimistic	**0.54** ^a^	0.41^a^	0.34^a^	0.33^a^	0.17^a^
GPH7	Confidence	**0.39** ^a^	0.07	0.14^b^	0.21^a^	0.06
GPH8	Fear	**0.51** ^a^	0.27^a^	0.35^a^	0.10	0.11
GPS1	Feeling low or sad	0.34^a^	**0.73** ^a^	0.49^a^	0.30^a^	0.37^a^
GPS2	Life being interesting	0.31^a^	**0.59** ^a^	0.44^a^	0.17^a^	0.15^a^
GPS3	Irritable	0.26^a^	**0.51** ^a^	0.24^a^	0.39^a^	0.47^a^
GPS4	Memory deterioration	0.27^a^	**0.55** ^a^	0.29^a^	0.28^a^	0.18^a^
GPS5	Health deterioration	0.11	**0.56** ^a^	0.27^a^	0.02	0.26^a^
GPS6	State of health	0.38^a^	**0.71** ^a^	0.57^a^	0.28^a^	0.24^a^
GPS7	Depression	0.21^a^	**0.53** ^a^	0.33^a^	0.21^a^	0.15^a^
GPS8	Disappointment	0.09	**0.56** ^a^	0.46^a^	0.06	0.17^a^
GPS9	Fear	0.43^a^	**0.70** ^a^	0.58^a^	0.32^a^	0.27^a^
GSO1	Social contact	0.39^a^	0.53^a^	**0.67** ^a^	0.22^a^	0.12
GSO2	Family relationship	0.29^a^	0.61^a^	**0.73** ^a^	0.06	0.09
GSO3	Friend relationship	0.36^a^	0.66^a^	**0.80** ^a^	0.17	0.17^a^
GSO4	Family support	0.02	0.29^a^	**0.10**	0.12	0.21^a^
GSO5	Other people’s care	0.13^b^	0.41^a^	**0.57** ^a^	0.23	0.34^a^
GSO6	Economic hardship	0.14^b^	0.48^a^	**0.69** ^a^	0.16	0.32^a^
GSO7	Labor status	0.19^a^	0.19^a^	**0.38** ^a^	0.11	0.10
GSO8	Family role	0.19^a^	0.47^a^	**0.70** ^a^	0.20	0.26^a^
GSS1	Nausea, vomiting	0.30^a^	0.18^a^	0.05	**0.47** ^a^	0.12
GSS2	Lose hair	0.26^a^	0.07	0.02	**0.61** ^a^	0.16^b^
GSS3	Oral ulcer	0.07	0.02	0.01	**0.29** ^a^	0.14^b^
GSS4	Pain	0.38^a^	0.29^a^	0.24^a^	**0.68** ^a^	0.54^a^
GSS5	Thin	0.30^a^	0.29^a^	0.20^a^	**0.64** ^a^	0.38^a^
GSS6	Dry mouth tastes bitter	0.26^a^	0.19^a^	0.12	**0.60** ^a^	0.23^a^
GSS7	Fatigue	0.30^a^	0.32^a^	0.22^a^	**0.69** ^a^	0.46^a^
SBR1	Breast distending pain	0.17^a^	0.38^a^	0.19^a^	0.37^a^	**0.72** ^a^
SBR2	Activity limitation	0.19^a^	0.23^a^	0.12	0.37**^a^**	**0.62** ^a^
SBR3	Upper limb pain	0.11	0.27^a^	0.17^a^	0.38^a^	**0.69** ^a^
SBR4	Abnormal breast skin	0.10	0.31^a^	0.10	0.37^a^	**0.69** ^a^
SBR5	Abnormal lump	0.02	0.36^a^	0.25^a^	0.08	**0.54** ^a^
SBR6	Body image	0.05	0.13^b^	0.15^b^	0.05	**0.44** ^a^
SBR7	Sexual life	0.06	0.15^a^	0.15^b^	0.15	**0.45** ^a^
SBR8	Muscles and joints being sore	0.21^a^	0.08	0.02	0.32a	**0.45** ^a^
SBR9	Chest tightness	0.19^a^	0.23^a^	0.02^a^	0.37^a^	**0.47** ^a^
SBR 10	Bone pain	0.08	0.09	0.60^a^	0.40^a^	**0.39** ^a^

The correlations between each item and its designated scale are in bold. ^a^Significant at the level of 0.01. ^b^Significance at the level of 0.05.

In this study, an exploratory factor analysis was carried out on the general and the specific module of the scale, and the results showed that the KMO of the general module and the specific module are 0.819 and 0.752, respectively. There is a strong partial correlation between variables, and Bartlett’s spherical test for both was *P <*0.001, suggesting that the variables are not independent of each other and that factor analysis is suitable for data analysis.

In factor analysis of the general module, the principal component method is used to extract the common factors whose characteristic roots are greater than 1. Ten principal components were extracted with the cumulative contribution rate of variance being 71.29%. After maximum variance rotation, it can be seen that the construct of the general module set by the extracted principal components is basically consistent with the original theoretical assumption.

In factor analysis of the specific module, the principal component method is used to extract the common factors whose characteristic roots are greater than 1. Three principal components are obtained, and the cumulative contribution rate of the variance is 64.17%. After maximum variance rotation, it can be seen that the characteristic root of the first principal component is 3.24, which mainly reflects the related symptoms of breast cancer, involving items SBR1, SRB2, SBR3, SBR4, and SBR5, and the variance contribution rate is 32.41%. The second principal component characteristic root is 1.70, which mainly reflects the side effects of disease treatment prognosis, involving items SBR8, SBR9, and SBR10, and the variance contribution rate is 17.01%. The characteristic root of the third principal component is 1.47, which mainly reflects the unique psychological changes of breast cancer patients, involving items SBR6 and SBR7, and the variance contribution rate is 14.74%. It is basically consistent with the breast cancer-specific module framework proposed in advance.

From the results mentioned above, theoretical construct was confirmed by data analysis, and good construct validity was shown.

The correlation coefficients between the QLICP-BR V2.0 and FACT-B (V4.0) domain scores indicate that the correlation between the same and similar domains (bold in the table) is usually higher than that with different or dissimilar domains—for example, the correlation coefficients between the physiological status domain (PWB) and functional status domain (FWB) of the FACT-B (V4.0) and the PHD of the QLICP-BR V2.0 are 0.39 and 0.44, respectively. The correlation coefficients between the emotional status (EWB) and functional status (FWB) domains of the FACT-B (V4.0) and the PSD of the QLICP-BR V2.0 are 0.60 and 0.67, respectively. The correlation coefficient between the emotional status (EWB) of the FACT-B (V4.0) and the SOD of the QLICP-BR V2.0 is 0.59. On the other hand, the correlation coefficients between the additional focus domain (AC) of the FACT-B (V4.0) and the SSD and SPD of the QLICP-BR V2.0 are 0.61 and 0.48, respectively.

#### 3.2.3 Responsiveness

The data from 246 patients who completed the questionnaire after treatments were used to assess responsiveness. The paired *t*-test and the response index SRM were used to check the average score change of each domain/facet of QLICP-BR V2.0 before and after treatments. The results are shown in **Table 3**. It can be seen that all domains/facets and overall scale have undergone major changes (*P* < 0.01). The SRM of the total scale is 0.61, and the SRM of all the domains are greater than 0.40, with the exception of PHD. It can be considered that QLICP-BR V2.0 scales have good responsiveness.

**Table 3:** Responsiveness of the quality-of-life instrument QLICP-BR (V2.0) (*n* = 246).

QLICP-BR	Before treatmentMean SD	After treatmentMean SD		DifferencesMean SD			*t*	*p*	SRM
Physical domain	**70.24**	**11.42**	**77.43**	**16.27**	**7.44**	**19.22**	**5.894**	**0.000**	**0.37**
Basic physiologic	65.26	13.11	75.97	17.51	11.31	20.11	8.572	0.000	0.53
functions									
Mobility and mobility	78.52	17.14	79.89	18.25	0.97	23.40	0.631	0.529	0.06
Psychological domain	**69.48**	**12.92**	**76.82**	**15.66**	**7.14**	**15.55**	**6.992**	**0.000**	**0.47**
Cognition	78.91	15.30	83.24	14.94	3.83	17.63	3.306	0.000	0.25
Emotion	68.03	14.79	75.02	17.18	6.75	16.29	6.309	0.000	0.43
Will and personality	63.67	18.43	74.89	20.14	11.42	23.58	7.377	0.000	0.48
Social domain	**64.89**	**10.43**	**71.55**	**17.07**	**6.20**	**16.13**	**5.850**	**0.000**	**0.41**
Interpersonal	69.72	12.68	74.41	19.19	4.26	18.94	3.424	0.001	0.25
Communication									
Social support and security	63.16	9.94	69.72	23.02	6.33	23.49	4.105	0.000	0.28
Social role	63.52	16.45	72.36	22.05	7.87	18.90	6.339	0.000	0.47
Common symptoms	**83.96**	**13.17**	**90.41**	**12.47**	**6.07**	**15.58**	**5.929**	**0.000**	**0.41**
and side effects									
Common symptoms	80.66	19.07	89.19	13.59	7.72	20.72	5.678	0.000	0.41
Common side effects	86.43	14.13	91.33	14.20	4.82	15.92	4.613	0.000	0.31
QLICP-GM	**71.92**	**8.49**	**78.32**	**12.75**	**6.26**	**13.17**	**7.245**	**0.000**	**0.49**
Specific domain	**83.26**	**12.16**	**90.46**	**10.76**	**6.62**	**12.01**	**8.393**	**0.000**	**0.60**
Clinical symptoms	85.26	17.96	92.97	10.37	6.47	15.89	6.196	0.000	0.49
Treatment side effects	90.89	12.50	94.61	9.85	3.52	12.36	4.336	0.000	0.30
Specific psychological effects	66.82	23.60	77.96	25.05	11.64	23.22	7.635	0.000	0.48
Total	**73.20**	**8.70**	**81.07**	**13.25**	**7.56**	**12.82**	**8.979**	**0.001**	**0.61**

Bold values represent results for domains of the scale. Other values represent results for facets of domains.

### 3.3 Evaluation Results of Modern Measurement Theory

#### 3.3.1 G-Study Results

In the PHD, PSD, SOD, SSD, and SPD domains, the variation components of the interaction between the subject and the item are 0.624, 0.558, 0.521, 0.670, and 0.626, respectively. The variance components of the subjects in the five domains are between 0.109 and 0.205, and the variance components of the items are between 0.121 and 0.253, indicating that the variance of the scale score is mainly due to the interaction between the subject and the scale items, and other factors lead to the smaller variance in the patients’ scale scores. Detailed results are shown in **Table 4**.

**Table 4:** Estimation of variance and covariance components in various domains in the *p* ^•^× *i* ^°^-designed G-study (*n* = 246).

	PHD	PSD	SOD	SSD	SPD
*P*	**0.131**	0.617	0.596	0.732	0.263
	0.101	**0.205**	0.970	0.468	0.543
	0.071	0.145	**0.109**	0.303	0.359
	0.113	0.090	0.043	**0.182**	0.720
	0.040	0.102	0.049	0.128	**0.174**
I	**0.207**				
		**0.253**			
			**0.121**		
				**0.127**	
					**0.149**
P*i	**0.624**				
		**0.558**			
			**0.521**		
				**0.670**	
					**0.626**

The elements on the main diagonal are the estimates of the variance components of each effect in the corresponding fields (shown in bold), the elements below the main diagonal are the estimates of the covariance components of the effects in different fields, and the elements above the main diagonal are the correlation coefficients between each field. PHD, physical domain; PSD, psychological domain; SOD, social domain; SSD, common symptoms and side effect domain; SPD, specific domain.

#### 3.3.2 D-Study Results

According to D-study, the generalization coefficients (*G* coefficient) of scores in the 5 domains are between 0.626 and 0.768, the reliability index (*Ф* coefficient) is between 0.557 and 0.695, and all domains fluctuate around 0.6. The relative error variance is within 0.1, and the absolute error variance is within 0.2, indicating that the reliability of these five domains is relatively high, which is basically consistent with the results of the classical test theory cited above.

In the case of 10 items of breast cancer-specific modules, the generalization coefficient and the reliability index of the specific module are 0.735 and 0.692, respectively. When the number of patients is fixed and the general module remains the same, the number of items increases from 5 to 15, the absolute error and the relative error decrease in sequence, and the generalization coefficient and the reliability index increase in sequence. The results of the D-study of QLICP-BR V2.0 are shown in **Table 5**.

**Table 5:** *P* ^•^× *I* ^°^- designed D-study results of the various domains of QLICP-BR V2.0.

Index	PHD	PSD	SOD	SSD	SPD				
	(*n* = 8)	(*n* = 9)	(*n* = 8)	(*n* = 7)	(*n* = 5)	(*n* = 8)	(*n* = 10)	(*n* = 12)	(*n* = 15)
$\sigma_{P}^{2}$	0.131	0.205	0.109	0.182	0.174	0.174	0.174	0.174	0.174
$\sigma_{\delta }^{2}$	0.078	0.062	0.065	0.096	0.125	0.078	0.063	0.052	0.042
$\sigma_{\Delta }^{2}$	0.104	0.090	0.080	0.114	0.155	0.097	0.078	0.065	0.052
$\sigma_{X_{PI}}^{2}$	0.027	0.029	0.016	0.019	0.031	0.020	0.016	0.013	0.011
*G*	0.626	0.768	0.626	0.655	0.581	0.690	0.735	0.769	0.806
*Ф*	0.557	0.695	0.576	0.615	0.529	0.642	0.692	0.729	0.771

$\sigma_{P}^{2}$ , global score variance; $\sigma_{\delta }^{2}$ , relative error variance; $\sigma_{\Delta }^{2}$ , absolute error variance; $\sigma_{X_{PI}}^{2}$ , use the sample mean to estimate the error variance of the global score; G, generalization coefficient; Ф, reliability index; PHD, physical domain; PSD, psychological domain; SOD, social domain; SSD, common symptoms and side effect domain; SPD, specific domain. ^a^All values in this column are composite values of indicators such as $\sigma_{Pc}^{2}$ , $\sigma_{\delta c}^{2}$ , $\sigma_{\Delta c}^{2}$ , G_c_, and Ф_c_.

## 4 Discussions

### 4.1 Scale Development

This study is mainly aimed at breast cancer patients, which is part of the cancer patient reporting outcome measurement scale system. By learning from the experience in the development of mature scales at home and abroad, adopting the advanced model of combining general and specific modules in structure, following the rules and procedures of scale formulation, and combining with the actual situation in China, QLICP-BR V2.0 was developed.

### 4.2 Scale Evaluation

In this study, the test–retest reliability, Cronbach coefficient, and ICC have been calculated to confirm good reliability. In addition, the item–domain correlation analysis, exploratory factor analysis, FACT-B (V4.0) as a criterion to calculate the criterion-related validity, *etc.*, have confirmed that the scale has good validity. Furthermore, the paired *t*-test and the calculation of SRM indicators confirm that the scale has a good degree of responsiveness. The results of the GT of this scale show that the main source of scale score variation is the interaction between patients and items. The purpose of this scale is to measure the quality of life of patients, so the source of variation in the scale is more reasonable. In the D-study of this research, the *G* and *Ф* coefficients for the current number of items and the recommended number of items according to the *G* and *Ф* coefficients after fixing the subjects are also presented. The standard for *G* and *Ф* coefficients is 0.6. When these two indicators are greater than 0.6, the scale is considered reliable. According to **Table 5**, this study shows that, when the number of specific module entries is 5, it is not satisfactory. As the number of items increases, the *G* and *Ф* coefficients are increasing, but when the number of items is greater than 10, the magnitude of the increase begins to decrease. Therefore, the number of items of the specific module was finally determined to be 10 (28).

The development of QLICP-BR V2.0 is based on QLICP-BR (V1.0). In terms of psychometric characteristics, the second version of the scale adopts FACT-B as the criterion scale. Compared with the first version (QLQ-BR53 as the criterion scale), the division of domains in QLICP-BR V2.0 is clearer, the correlation coefficient in the same domain is larger, and it has better criterion-related validity. In addition, the responsiveness of the second version of the scale is more obvious, and it is statistically significant in all domains when evaluating the effect of the treatment plan, while the first version of the scale is not the case. Finally, the QLICP-BR V2.0 development process adopted the multivariate generalization theory, which is an indicator of measurement reliability developed by organically integrating true score theory and variance analysis. The random error is further divided into different source components, their respective proportions are examined, and their indicators are calculated to reflect the accuracy and stability of the measurement results. The G-study can randomly sample from a clearly defined range, and it is not limited by observable results, and can provide evidence of validity based on the test content (29).

### 4.3 Clinical Application and Related Research

The QLICP-BR V2.0 scale can be applied in many aspects of clinical research, such as evaluating the effectiveness of treatment measures and the feasibility of intervention programs, exploring factors affecting the quality of life in breast cancer, accurately capturing changes in patients’ symptoms, evaluating and improving the quality of medical services, optimizing benefit evaluation of health resource investment, *etc.* (30). In the 1970s, the MAPI Institute in Lyon, France, established the PROQOLID database, which used the network to push the patient-reported outcome scale and the quality-of-life scale to the relevant medical staff (31). Fisher *et al.* conducted a 20-year follow-up survey of breast cancer patients undergoing mastectomy and breast-conserving surgery and found that women who underwent breast-conserving surgery were more satisfied with their body image and had better functional status and fewer symptoms (32, 33).

There are also some related studies on breast cancer scales at home and abroad, such as EORTC QLQ-BR53 (QLQ-C30 and QLQ-BR23), FACT-B, SLDS-BC, BIBCQ, HIBS, BREAST-Q, BCTOS, and so on, but they are lacking Chinese cultural backgrounds to some extent considering their original use in English-speaking patients. Some scales, such as those specific to breast cancer surgery, have not been rigorously assessed (7).

### 4.4 Limitations of the Present Study

In this study, some improvements are necessary before QLICP-BR V2.0 can be used as a practical instrument to measure and assess the QOL of Chinese breast cancer patients. The survey of this study is limited to inpatients. In the future, the survey should be extended to outpatient or community patients, and IRT methods should be used to obtain more representative survey results.

## 5 Conclusion

Given what has been discussed above, QLICP-BR V2.0 exhibited reasonable degrees of validity, reliability, and responsiveness according to classical test and generalizability theories.

## Data Availability Statement

The original contributions presented in the study are included in the article/supplementary material. Further inquiries can be directed to the corresponding author.

## Ethics Statement

The study protocol and the written informed consent form were approved by the IRB (institutional review board) of the affiliated hospital of Guangdong Medical University (PJ2012052, YJYS2019010). The respondents were voluntary and provided written informed consent for participation.

## Author Contributions

CW designed the study. FL, ZY, JZ, QL, WL, and HC performed the data collection. FL performed the data analyses and drafted the manuscript. CW revised the manuscript intensively. All authors contributed to the article and approved the submitted version.

## Funding

This study is supported by the National Natural Science Foundation of China (71974040 and 81273185) and the Features Innovative Projects of Key Platform and Major Scientific Research Project of Universities in Guangdong Province (2017KZDXM040 and 2018KZDXM037).

## Conflict of Interest

The authors declare that the research was conducted in the absence of any commercial or financial relationships that could be construed as a potential conflict of interest.

## Publisher’s Note

All claims expressed in this article are solely those of the authors and do not necessarily represent those of their affiliated organizations, or those of the publisher, the editors and the reviewers. Any product that may be evaluated in this article, or claim that may be made by its manufacturer, is not guaranteed or endorsed by the publisher.

## Abbreviations

QLICP-BR, Quality of Life Instruments system in breast cancer patients; QOL, quality of life; EORTC, European Organization for Research and Treatment; GT, Generalizability theory; CTT, classical test theory; MTT, modern test theory; IRT, item response theory; RS, raw scores; SS, standard score; SRM, standardized response mean; MGT, multivariate generalizability theory; UGT, univariate generalizability theory; CGD, core/general module; SPD, specific domain; TOT, total; ICC, intra-class correlation confident; SBR, specific module of breast cancer; PHD, physical domain; PSD, psychological domain; SOD, social domain; SSD, common symptoms and side effect domain; FWB, functional status domain; PWB, physiological status domain; EWB, emotional status; AC, additional focus domain; *G* coefficient, generalization coefficients; *Ф* coefficient, reliability index
